# Toxic Effects of Maternal Zearalenone Exposure on Intestinal Oxidative Stress, Barrier Function, Immunological and Morphological Changes in Rats

**DOI:** 10.1371/journal.pone.0106412

**Published:** 2014-09-02

**Authors:** Min Liu, Rui Gao, Qingwei Meng, Yuanyuan Zhang, Chongpeng Bi, Anshan Shan

**Affiliations:** Institute of Animal Nutrition, Northeast Agricultural University, Harbin, P. R. China; University of Arkansas for Medical Sciences, United States of America

## Abstract

The present study was conducted to investigate the effects of maternal zearalenone (ZEN) exposure on the intestine of pregnant Sprague-Dawley (SD) rats and its offspring. Ninety-six pregnant SD rats were randomly divided into four groups and were fed with diets containing ZEN at concentrations of 0.3 mg/kg, 48.5 mg/kg, 97.6 mg/kg or 146.0 mg/kg from gestation days (GD) 1 to 7. All rats were fed with mycotoxin-free diet until their offspring were weaned at three weeks of age. The small intestinal fragments from pregnant rats at GD8, weaned dams and pups were collected and studied for toxic effects of ZEN on antioxidant status, immune response, expression of junction proteins, and morphology. The results showed that ZEN induced oxidative stress, affected the villous structure and reduced the expression of junction proteins claudin-4, occludin and connexin43 (Cx43) in a dose-dependent manner in pregnant rats. Different effects on the expression of cytokines were also observed both in mRNA and protein levels in these pregnant groups. Ingestion of high levels of ZEN caused irreversible damage in weaned dams, such as oxidative stress, decreased villi hight and low expression of junction proteins and cytokines. Decreased expression of jejunal interleukin-8 (IL-8) and increased expression of gastrointestinal glutathione peroxidase (GPx2) mRNA were detected in weaned offspring, indicating long-term damage caused by maternal ZEN. We also found that the Nrf2 expression both in mRNA and protein levels were up-regulated in the ZEN-treated groups of pregnant dams and the high-dose of ZEN group of weaned dams. The data indicate that modulation of Nrf2-mediated pathway is one of mechanism via which ZEN affects gut wall antioxidant and inflammatory responses.

## Introduction

The global occurrence of mycotoxins constitutes a major risk factor for human and animal health, and an estimated 25% of the world's crop production is contaminated [1], [2]. Zearalenone (ZEN) is a non-steroidal estrogenic mycotoxin biosynthesized through a polyketide pathway by a variety of *Fusarium* fungi that are commonly found in feed and foodstuffs [3], [4]. It is frequently implicated in reproductive disorders of farm animals and occasionally in hyperoestrogenic syndromes of humans [5]. The adverse effects of ZEN may be even more pronounced during pregnancy, as the fetuses are susceptible to toxins due to their fragile developmental state and inadequate defense mechanism. Many studies have shown that ZEN can change the intrauterine environment during early gestation by affecting the secretory mechanism of the endometrium [6], [7]. Indeed, exposure of fetuses to ZEN led to impaired development and decreased litter size [8], [9]. It was demonstrated by Zhang, et al. that exposure to ZEN during early gestation affected maternal reproductive capability and delayed fetal development [10]. Likewise, many studies have shown that ZEN affects fecundity, and the effects could placental transfer to fetuses [11]–[13].

The intestinal tract is the first physical barrier against ingested food contaminants [14]. After ingestion of ZEN-contaminated food, enterocytes may be exposed to high concentrations of the toxin [15]. It has been demonstrated that ZEN easily crossed the intestinal barrier, and rapidly absorbed by enterocytes [16], [17]. Although xenobiotic biotransformation reactions occur mainly in the liver, the intestine may also contribute to overall biotransformation. At present, available data support that ZEN has hepatotoxic, haematotoxic, cytotoxic and genotoxic activities, which are not related to its binding affinity to estrogen receptor sites [18]–[21]. Furthermore, evidence of its effects on oxidative stress has emerged from several studies, which demonstrate that ZEN induces lipid peroxidation in the liver, spleen, kidney, and testis [22]–[25]. ZEN has also been shown to have immunotoxicity, which results in several alterations of immunological parameters [26]–[28]. Several studies *in vitro* have reported that ZEN induces cytotoxicity and oxidative damage, and inhibits protein and DNA syntheses in the human Caco-2 cell line [29], [30] or in the swine jejunal epithelial cells [31], [32]. However, to the best of our knowledge, the information *in vivo* that supports these observations is limited.

The epithelial surface consists of a simple columnar epithelium, which is increased by the presence of villi [33]. Intestinal mucosal permeability is closely related to the integrity of intestinal barrier. The function of the intestinal barrier is affected by its morphology and cellular junctions, including tight junctions and gap junctions [34]. The gut barrier is formed to a large extent by tight junctions containing occludin and one or more claudin isoforms [35]. Tight junctions seal the luminal end of intercellular space and limit transport via this paracellular route to relatively small hydrophilic molecules. In addition, gap junctions are a kind of structures that localized at the plasma membranes, and allow the exchange of ions, nucleotides, metabolites and other small molecules including second messengers between adjacent cells, thereby facilitating electrical and metabolic coupling [36], [37]. Gap junctions are clusters of transmembrane channels composed of connexin (Cx) dodecamers, of which the most widely expressed isoform is Cx43 [38], [39]. Based on its function as a physical barrier, intestine is also an active component of the immune system and creates a kind of barrier against invading pathogens [40], [41]. However, the increased intestinal barrier permeability may lead to intestinal inflammation [42].

The purpose of the present study was to investigate the effects of early pregnancy dietary exposure to different doses of ZEN on the intestine of the pregnant rats. We investigated the effects of ZEN on intestinal antioxidant status, cytokines expression, barrier function, and morphology in maternal rats. These indicators were also examined in weaned pups to test toxic effects of maternal ZEN exposure on intestinal development of offspring.

## Materials and Methods

### Ethics statement

This study was performed in strict accordance with the recommendations of the National Research Council Guide, and all of the animal experimental procedures were approved by the Ethical and Animal Welfare Committee of Heilongjiang Province, China. Rats were housed in a temperature-controlled room with proper darkness-light cycles, fed with a regular diet, and maintained under the care of the Laboratory Animal Unit, Northeast Agricultural University, China. All of the surgeries were performed through the ether anesthetization, and every effort was made to minimize suffering.

### Animals and feed treatments

Sprague-Dawley (SD) rats and basal diet used in this study were purchased from Jilin University Laboratory Animal Centre (Changchun, China). Ninety-six female rats weighing between 190 and 210 g and 24 male rats weighing between 300 and 325 g were prepared for the experiment. Male rats were used only as sires and were not subjected to any treatments. Rats were housed in multiple mouse racks and were acclimated for one week in groups of five per cage with free access to mycotoxin-free diet and tap water. The animal holding rooms were maintained on a 12-h light/12-h dark cycle at 24.5±0.5°C with 55±5% relative humidity.

After acclimation, the females were mated through naturally breeding (at a ratio of one male to two females). Every morning, each female was examined for the presence of sperm in the vaginal lavage. Each pregnant rat was maintained individually in a polycarbonate metabolic cage. The pregnant rats were randomized into four groups (ZEN0, ZEN50, ZEN100, and ZEN150), and were fed with diets containing ZEN at concentrations of 0.3 mg/kg, 48.5 mg/kg, 97.6 mg/kg or 146.0 mg/kg (equal to 0, 4.5, 9, and 13.5 mg/kg bw/d) respectively from gestation days (GD) 1 to 7. ZEN was purchased as pure crystals from Fermentek Ltd. (Jerusalem, Israel) and diluted in acetonitrile. The substance has been proven to be stable for at least eight months at room temperature. The doses applied in this study were selected on the basis of literatures reported by Ruddick et al. (1976), Collins et al. (2006) and Arora et al. (1981) [43]–[45].

### Sample collection

Twelve pregnant rats from each group reflecting the average body weight were selected and sacrificed by ether anesthetization on GD8. The remaining pregnant rats were fed with the standard mycotoxin-free diet until the pups were weaned at three weeks of age. Remaining twelve weaned dams and twelve pups with similar body weight selected randomly from each group were sacrificed by ether anesthetization. After slaughter, the jejunal sections were immediately excised and rinsed in normal saline. Afterwards, the samples were quickly dissected, frozen in the liquid nitrogen, and stored at −80°C until subjected to RNA extraction and Western blotting. Other sections of the jejunum were separated into two parts. One part was fixed in 10% formalin for morphological analysis and immunohistochemistry. The other part was sealed into pockets and preserved at −20°C until used for the evaluation of ZEN residues, antioxidant status, and cytokine synthesis.

### Detection of ZEN residues in the intestine

The jejunal tissues were analyzed for ZEN residues using ELISA kit. The ELISA method has been used for ZEN determination by Bennett et al. (1994) and Nuryono et al. (2005) [46], [47]. Jejunal samples of 0.5 g were homogenized with 2.5 ml of diluteapp: addword: dilute methanol (methanol: water  = 6∶4) and then extracted by shaking the mixture for 15 min. The sample solution was filtered through a folded paper filter, and the filtrate was diluted with the buffer solution provided in the kit. The sample solution was then treated according to the ZEN Kit, and absorbance was measured at 450 nm using an ultraviolet spectrophotometer.

### Assessment of lipid peroxidation

Jejunal tissues homogenates (10%) were prepared in chilled normal saline. The suspension was centrifuged at 3500 rpm for 10 min. Lipid peroxidation was determined by measuring the amounts of malondialdehyde (MDA) through the thiobarbituric acid method described by Bloom and Westerfe (1971) using a commercial MDA kit [48]. The absorbance was measured at 532 nm. Total proteins were quantified through the classical Bradford method with Coomassie Brilliant Blue G-250. Concentration of MDA was expressed as nmol/mg of protein.

### Evaluation of antioxidant enzyme activity

The activities of total superoxide dismutase (SOD), glutathione peroxidase (GPx) and catalase (CAT) were analyzed using commercial reagent kits [49]. Analysis of the SOD activity was based on the SOD-mediated inhibition of the formation of nitrite from hydroxylammonium in the presence of O^2−^ generators (xanthine/xanthine oxidase) [50]. Activity of SOD was expressed as units/mg of protein and determined by measuring the reduction of in the optical density of the reaction solution at 550 nm. GPx is an enzyme that catalyzes glutathione oxidation by oxidizing the reduced tripeptide glutathione (GSH) into oxidized glutathione [51]. GPx activity was expressed as units/mg of protein, and one unit was defined as the amount required to decrease the GSH by 1 mM/min after subtracting the decrease in GSH per minute obtained with the nonenzymatic reaction. CAT activity was assayed by the method developed by Aebi (1984) [52], and calculated as nM H_2_O_2_ consumed/min/mg of tissue protein.

### Cytokine synthesis

Jejunal tissue homogenates (10%) were analyzed for cytokines content by sandwich ELISA. Commercial reagent kits were used to detected the interleukin (IL)-1α, IL-1β, IL-6, IL-8, and tumor necrosis factor (TNF)-α (Shanghai, China). A purified fraction of anti-rat cytokines were used as the capture antibodies in conjunction with biotinylated anti-rat cytokines. Streptavidin-HRP and tetramethylbenzidine were used for the detection. The absorbance was read at 450 nm using a microplate reader. Recombinant rat IL-1α, IL-1β, IL-6, IL-8, and TNF-α were used as standards, and results are expressed as nanograms of cytokine/mL. All of the tests were performed using four independent replicates.

### Real time-PCR (RT-PCR)

Total RNA was extracted from approximately 100 mg of frozen jejunal tissues using the reagent box of Total RNA Kit, according to the manufacturer's instructions. The concentration of RNA was measured by using a spectrophotometer, and the purity was ascertained by the *A260/A280* ratio. Total RNA from each sample was converted into cDNA according to the manufacturer's instructions and used for RT-PCR.

SYBR Green I RT-PCR Kit was used to measure mRNA expression of antioxidant genes (Nrf2 and gastrointestinal GPx (GPx2)), tight junctions (occludin and claudin-4) and inflammatory cytokines (IL-1α, IL-1β, IL-6, IL-8, and TNF-α) expressed relative to the quantity of the β-actin endogenous control. Rat-specific primers were designed from published GenBank sequences and were synthesized by Sangon (Table 1).

**Table 1:** Nucleotide sequences of primers for RT-PCR.

Gene	Forward primer(from 5′ to 3′) Reverse primer(from 5′ to 3′)	Fragment length (bp)	Genbank no.
Nrf2	CTGCTGCCATTAGTCAGTCG	101	NM_031789.2
	GCCTTCAGTGTGCTTCTGGT		
GPx2	CCGTGCTGATTGAGAATGTG	113	NM_183403.2
	AGGGAAGCCGAGAACCACTA		
occludin	CTCCAACGGCAAAGTGAATG	104	NM_031329.2
	CGGACAAGGTCAGAGGAATC		
claudin-4	CTGCCTGGAGTCTTGGTGTC	122	NM_001012022.1
	GAGGGTAGGTGGGTGGGTAA		
IL-1β	GCCAACAAGTGGTATTCTCCA	120	NM-031512.2
	TGCCGTCTTTCATCACACAG		
IL-1α	GAGTCGGCAAAGAAATCAAGA	112	NM_017019.1
	TTCAGAGACAGATGGTCAATGG		
IL-6	AGTTGCCTTCTTGGGACTGA	102	NM_012589.2
	ACTGGTCTGTTGTGGGTGGT		
IL-8	AAGAGGGCTGAGAACCAAGA	124	XM_004833923.1
	CCCACACAATACACAAAGAACTG		
TNF-α	TTCCGTCCCTCTCATACACTG	149	NM_012675.3
	AGACACCGCCTGGAGTTCT		
β-actin	ACCCGCGAGTACAACCTTC	207	NM_031144
	CCCATACCCACCATCACACC		

For analyses on an ABI PRISM 7500 SDS thermal cycler, PCR reactions were performed with 2.0 µl of first-strand cDNA and 0.4 µl of sense and anti-sense primers in a final volume of 20 µl. Samples were centrifuged briefly and run on the PCR machine using the default fast program (1 cycle at 95°C for 30 s, 40 cycles of 95°C for 5 s and 60°C for 34 s). All of the PCR reactions were performed in triplicate. The relative gene expression levels were determined using the 2^−ΔΔCt^ method [53].

### Immunohistochemistry

Cx43 expression was analyzed on formalin-fixed, paraffin-embedded intestinal sections to evaluate intestinal-cell gap junctions. Tissue sections were deparaffinised with xylene and dehydrated through a graded ethanol series. Heat-mediated antigen retrieval was done by heating the sections (immersed in EDTA buffer, pH 8.0) in a microwave oven (750 W) for 15 min. Endogenous peroxidase activity was blocked by incubation in methanol-H_2_O_2_ solution. The tissue sections were quenched in normal goat serum for 30 min and incubated overnight at 4°C with the primary antibody (diluted 1∶75. Secondary antibody was applied followed by the streptavidin conjugated to horseradish peroxides. Finally, 3,3′-diaminobenzidine was used for color development, and hematoxylin was used for counter staining. The proportion of the intestinal section expressing Cx43 was estimated. Each sample was assessed as showing either normal or reduced staining. Normal staining was considered when homogeneous and strong basolateral membrane staining of the enterocytes were detected. Heterogeneous and weak staining were considered to indicate reduced expression.

### Western blotting

Total proteins in the intestinal tissues were extracted and the protein lysate were added. After schizolysis for 1 h on ice, the extracts were centrifugalization for 20 min in the speed of 12000 r/min at 4°C. Then the supernatant was taken, and Coomassie Brilliant Blue protein assay was used to detect the concentration of Nrf2 protein. Proteins (50 µg per lane) were transblotted to polyvinylidene difluoride membranes in standard Tris-glycine transfer buffer, pH 8.3, containing 0.5% SDS. After transfer, membranes were blocked for 1 h at room temperature in TBST (10 mmol/L Tris-HCl, pH 8.0, 150 mmol/L NaCl, 0.2% Tween-20) containing 5% non-fat milk powder, and incubated overnight at 4°C with diluted primary antibody (Nrf2) (1∶500 dilution). Membranes were then washed in TBST for three times. The second antibodies (1∶2000 dilution) labeled by horseradish peroxydase (Nrf2) were added, with incubation for 2 h at 37°C and washing the membrane for three times. To demonstrate equal loading, membranes were stripped and reprobed with a specific antibody recognizing β-actin. Band densities were obtained by scanning the membranes using the Quantity One software. Density data were standardised within membranes by expressing the density of each band of interest relative to that of β-actin in the same lane.

### Morphological analysis

Cross-sectional jejunal samples from the formalin-preserved segments were cut into 2-mm^2^ sections and fixed by standard paraffin embedding. Samples were sectioned at 5 µm and stained with hematoxylin eosin. Each slide was divided into three single segments and analyzed the microstructures of jejunum using an optical microscope (Nikon Eclipse E400). The villous heights and crypt depths of 30 randomly chosen villi were measured.

### Statistical analysis of data

The indices were analyzed through ANOVA and Duncan's multiple range tests using the SPSS19.0 statistical software. The data are expressed as the means ± standard error of the mean (mean ±SEM). The level of significance was accepted as P<0.05.

## Results

### ZEN induces oxidative stress in the jejunum

**Figure 1:**
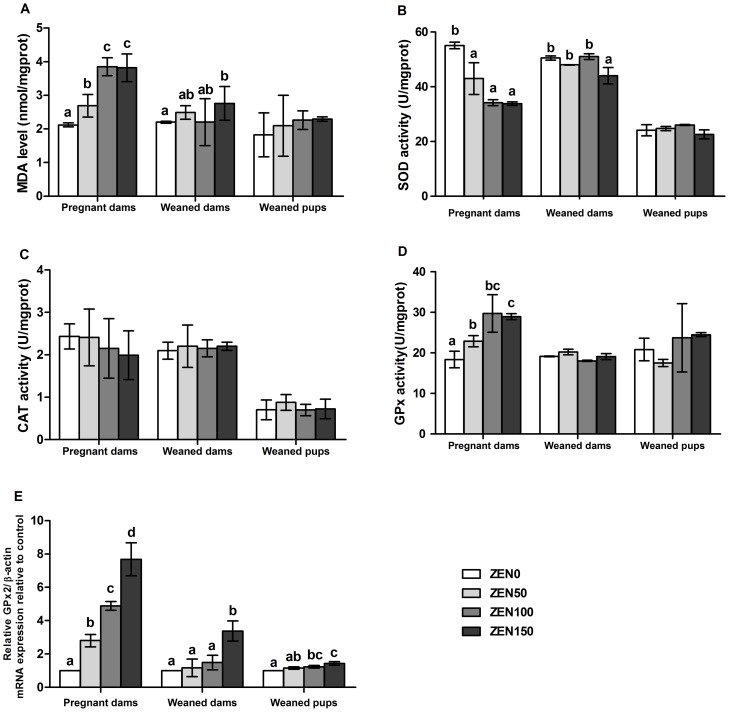
Effects of zearalenone (ZEN) on oxidative stress in the jejunum of rats. (A) malondialdehyde (MDA) level; (B) total superoxide dismutase (SOD) activity; (C) catalase (CAT) activity; (D) glutathione peroxidase (GPx) activity; (E) intestinal glutathione peroxidase (GPx2) mRNA expression. The relative gene expression for GPx2 was calculated relative to the gene expression of the ZEN0 group. β-actin was chosen as a reference gene having the same relative expression mean in the ZEN0 group as in the treated groups. ZEN 0, ZEN 50, ZEN 100, and ZEN 150 means a daily feeding with ZEN at doses of 0.3, 48.5, 97.6, and 146.0 mg/kg respectively, at gestation days 1 to 7. All of the values are expressed as the means ±SEM (n = 12). ^a, b, c^ means within a row with no common superscripts differ significantly (P<0.05).

ZEN induced a significant increase of MDA formation in the ZEN-treated groups of pregnant dams and the ZEN150 group of weaned dams (P<0.05). The MDA concentrations in the offspring exhibited an increasing tendency, but this difference was not significant (P>0.05). Dietary ZEN reduced the activity of SOD in the jejunum of pregnant dams, and the significantly decreased activity of SOD was also observed in the ZEN150 group of weaned dams (P<0.05). We also found that the MDA concentrations and SOD activity were not different between the ZEN100 and ZEN150 group of pregnant dams. The activity of CAT in the pregnant dams decreased in a dose-dependent manner, but this difference was not significant (P>0.05). Significant increases both in GPx activity and GPx2 mRNA were seen in all ZEN-treated groups of pregnant dams (P<0.05). GPx2 mRNA expression was also up-regulated in the ZEN150 group of weaned dams and in the ZEN100 and ZEN150 groups of weaned pups (P<0.05). The jejunal extracts of pups showed normal levels in CAT, SOD and GPx activity compared with the ZEN0 group (Figure 1).

### ZEN alters cytokines both in protein and mRNA levels of the jejunum

We investigated the effects of different doses of ZEN on cytokine secretion involved in the inflammatory response in the intestinal samples. Pregnant dams exposed to ZEN50 exhibited increased IL-1α and decreased TNF-α synthesis (P<0.05). Both the IL-1β and TNF-α synthesis were down-regulated in the ZEN150 group of pregnant dams (P<0.05). Although IL-6 and IL-8 synthesis had a decreased tendency in the ZEN-treated groups, the differences were not statistically significant (P>0.05). No negative effects were observed on the cytokines concentrations in the weaned dams. However, the concentration of IL-8 in the offspring exhibited a significant decrease in the ZEN100 and ZEN150 groups (P<0.05) (Table 2).

**Table 2:** Effect of zearalenone (ZEN) on the cytokine synthesis on intestine in pregnant dams at gestation day 8, and weaned dams and pups at three weeks after born (ng/L).

	ZEN0	ZEN 50	ZEN 100	ZEN 150
Pregnant dams				
IL-1β	30.51±0.01^b^	29.88±0.10^b^	30.00±0.30^b^	28.68±0.66^a^
IL-1α	33.467±0.03^b^	34.50±0.36^a^	33.81±0.12^b^	33.26±0.39^b^
IL-6	87.46±0.08	87.32±0.85	87.33±1.99	87.00±1.80
IL-8	315.35±4.79	314.80±6.84	312.43±6.84	312.42±0.01
TNF-α	259.65±2.50^c^	253.28±1.05^b^	257.83±1.58^c^	245.87±1.50^a^
Weaned dams				
IL-1β	26.99±0.03	26.83±0.82	27.16±0.88	27.22±0.51
IL-1α	33.94±0.48	34.08±0.04	33.12±0.39	33.41±0.77
IL-6	84.53±0.11	81.08±1.23	81.74±1.04	81.32±0.66
IL-8	276.85±0.99	288.71±2.74	288.12±5.82	291.38±6.29
TNF-α	247.81±0.31	238.71±6.31	237.52±0.41	241.25±5.27
Weaned pups				
IL-1β	4.73±0.09	4.78±0.09	4.66±0.07	4.69±0.05
IL-1α	27.52±0.29	27.54±0.65	26.83±0.51	27.99±0.41
IL-6	65.74±1.31	65.17±1.38	65.99±2.36	65.26±2.29
IL-8	253.74±5.12^bc^	257.76±2.53^c^	245.05±4.03^b^	230.62±0.67^a^
TNF-α	198.44±2.51	200.11±3.19	196.42±4.22	193.62±2.20

All of the values are expressed as the means ±SEM (n = 12).

ZEN 0, ZEN 50, ZEN 100, and ZEN 150 means a daily feeding with ZEN at doses of 0.3, 48.5, 97.6, and 146.0 mg/kg respectively, at gestation days 1 to 7.

a, b, c means within a row with no common superscripts differ significantly (P<0.05).

**Figure 2:**
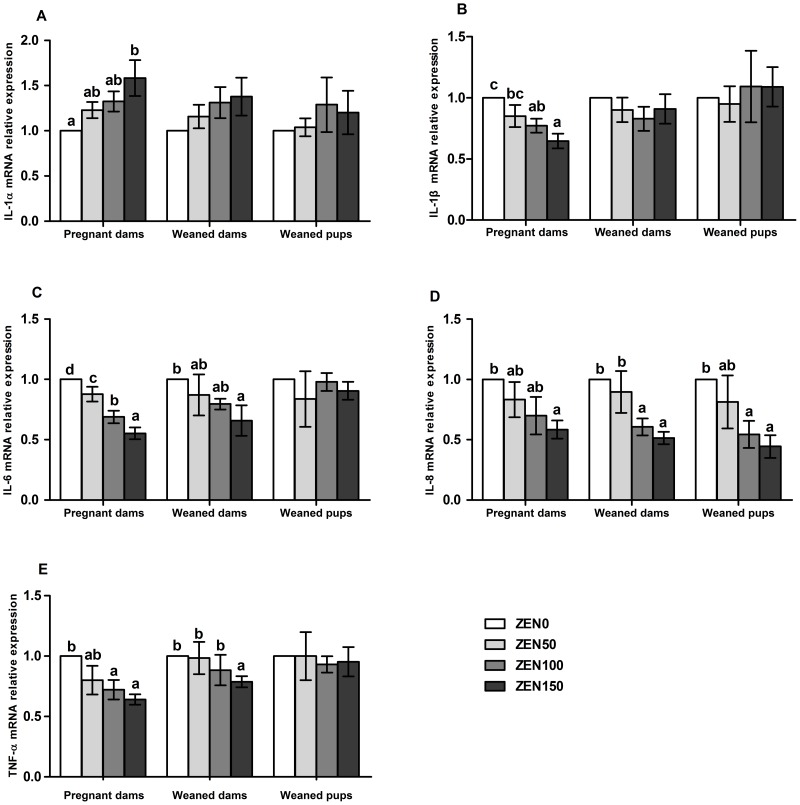
Effects of zearalenone (ZEN) on the expression of immune genes in the jejunum of rats. (A) Interleukin (IL)-1α; (B) IL-1β; (C) IL-6; (D) IL-8; (E) Tumor necrosis factor (TNF)-α. The relative gene expression for each gene was calculated relative to the gene expression of the ZEN0 group. β-actin was chosen as a reference gene having the same relative expression mean in the ZEN0 group as in the treated groups. ZEN 0, ZEN 50, ZEN 100, and ZEN 150 means a daily feeding with ZEN at doses of 0.3, 48.5, 97.6, and 146.0 mg/kg respectively, at gestation days 1 to 7. All of the values are expressed as the means ±SEM (n = 12). ^a, b, c^ means within a row with no common superscripts differ significantly (P<0.05).

We further quantified the expression of genes coding for cytokines, using RT-PCR. Significantly increased IL-1α mRNA and decreased IL-1β, IL-6, IL-8 and TNF-α mRNA were observed in the ZEN-treated groups of pregnant dams with a dose-dependent manner (P<0.05). For the weaned dams, the IL-6 (ZEN150), IL-8 (ZEN100, ZEN150) and TNF-α (ZEN150) mRNA were down-regulated (P<0.05). For the weaned pups, the IL-8 mRNA was also down-regulated in the ZEN100 and ZEN150 groups (P<0.05) (Figure 2).

### ZEN up-regulates the expression of Nrf2 in the jejunum

**Figure 3:**
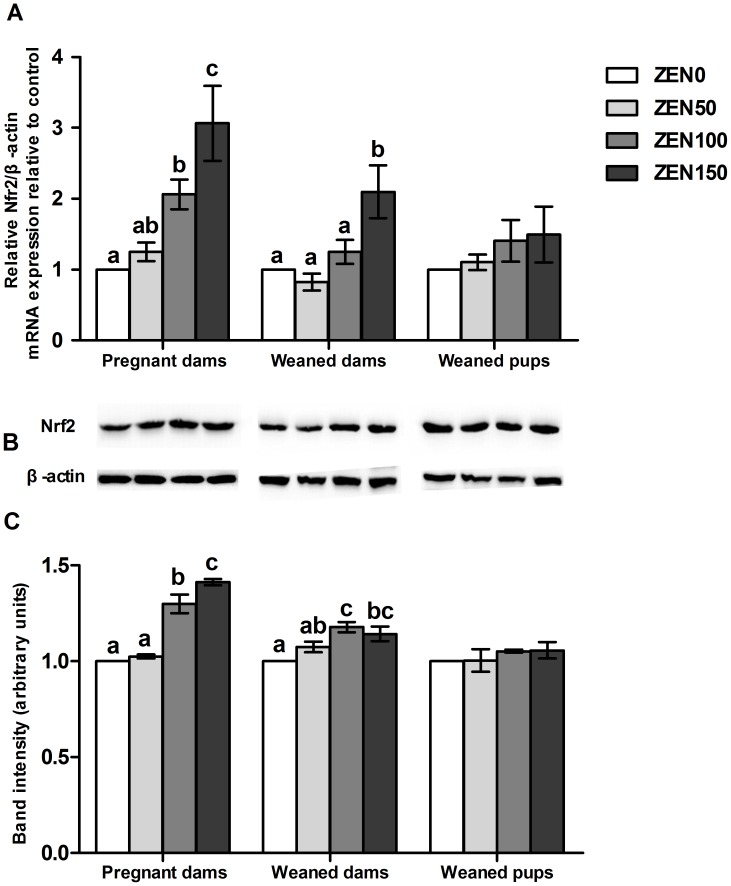
Effects of zearalenone (ZEN) on the expression of Nrf2 in jejunum of rats. (A) Nrf2 mRNA expression; (B) The immunoblot; (C) The expression of the Nrf2 protein estimated by densitometric analyses after normalisation with the β-actin signal. ZEN 0, ZEN 50, ZEN 100, and ZEN 150 means a daily feeding with ZEN at doses of 0.3, 48.5, 97.6, and 146.0 mg/kg respectively, at gestation days 1 to 7. All values are expressed as mean ±SEM (n = 6). ^a, b, c^ means within a row with no common superscripts differ significantly (P<0.05).

The results of RT-PCR and Western blotting showed that the expression of Nrf2 was increased in pregnant dams in a dose-dependent manner, and the changes were significant in ZEN100 and ZEN150 groups (P<0.05). In weaned dams, a significant up-regulation of Nrf2 mRNA was observed in the ZEN150 group, while the protein levels were increased in ZEN100 and ZEN150 groups (P<0.05). Nrf2 expression was not affected in other groups (Figure 3).

### ZEN reduces mRNA expression of tight junction proteins

**Figure 4:**
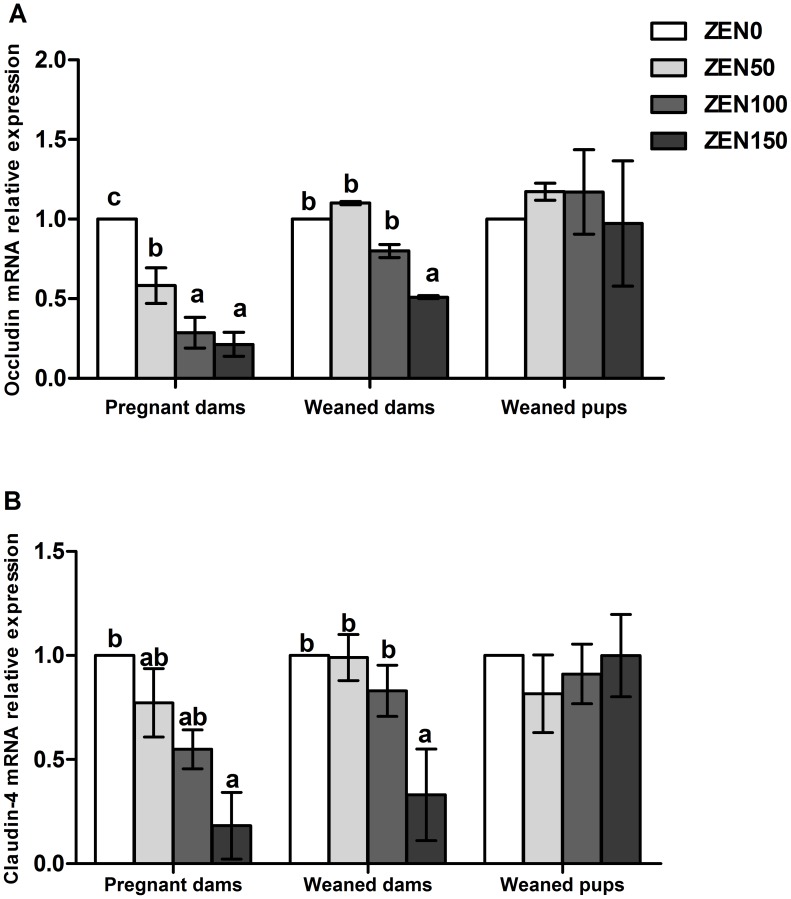
Effects of zearalenone (ZEN) on the mRNA expression of tight junctions in the jejunum of rats. (A) occludin; (B) claudin-4. The relative gene expression for each gene was calculated relative to the gene expression of the ZEN0 group. β-actin was chosen as a reference gene having the same relative expression mean in the ZEN0 group as in the treated groups. ZEN 0, ZEN 50, ZEN 100, and ZEN 150 means a daily feeding with ZEN at doses of 0.3, 48.5, 97.6, and 146.0 mg/kg respectively, at gestation days 1 to 7. All values are expressed as mean ±SEM (n = 12). a, b, c means within a row with no common superscripts differ significantly (P<0.05).

Effects of ZEN on the expression of the tight junction proteins, including occludin and claudin-4, were assessed using RT-PCR. Both the occludin and claudin-4 mRNA expression levels in pregnant dams showed a declining trend with increase of ZEN. Expression of the occludin mRNA in the ZEN100, ZEN150 groups and the claudin-4 mRNA in the ZEN150 group were significantly down-regulated in pregnant dams (P<0.05). The ZEN150 group still exhibited low occludin and claudin-4 mRNA expression levels in weaned dams (P<0.05). ZEN had no significant effects on the mRNA expression of the occludin and claudin-4 in the offspring (P>0.05) (Figure 4).

### ZEN decreases the expression of the gap-junction Cx43 in the jejunum

**Figure 5:**
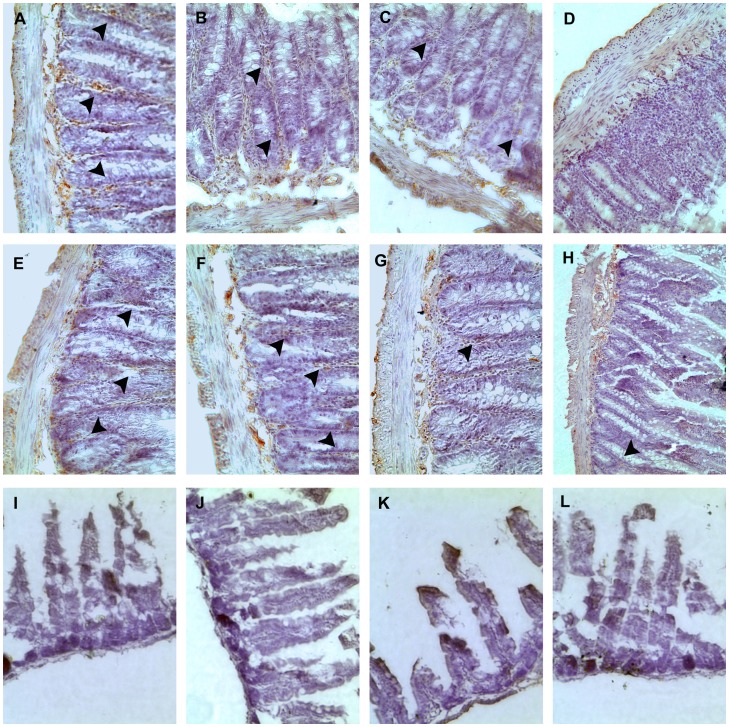
Effect of zearalenone (ZEN) on the expression of gap-junction Cx43 in rats. Samples were collected from the pregnant dams (A–D) at gestation day 8, and weaned dams (E–H) and pups (I–L) at three weeks after born. The results were showed from left to right-ZEN0, ZEN50, ZEN100, ZEN150 in each period of the rats. (A–H) Cx43 immunoreactivity appeared along the smooth muscle surface both in the outer circular layer and innermost circular layer of the jejunal samples. (A–D) The Cx43 immunoreactivity observed in the intestinal glands was down-regulated in the ZEN-treated groups of pregnant dams (arrow). (E–H) the weak immunoreactivity of Cx43 was also observed in the ZEN150 group. (I–L) less Cx43 immunoreactivity was observed. 400×. ZEN 0, ZEN 50, ZEN 100, and ZEN 150 means a daily feeding with ZEN at doses of 0.3, 48.5, 97.6, and 146.0 mg/kg respectively, at gestation days 1 to 7. ^a, b, c^ means within a row with no common superscripts differ significantly (P<0.05).

Cx43 immunoreactivity appeared along the smooth muscle surface both in the outer circular layer and innermost circular layer of the jejunum in dams. Strongly positive immunoreactivity results were also obtained in the intestinal glands (arrow). Significant and dose-related decreases in Cx43 immunoreactivity were seen in all of the ZEN-treated groups of pregnant dams. In the ZEN150 group, low Cx43 immunoreactivity was also observed in weaned dams. There was less obvious Cx43 immunoreactivity in the jejunum of the weaned pups, and the difference was not significant from the ZEN0 group (Figure 5).

### ZEN alters villous structure in the jejunum

**Figure 6:**
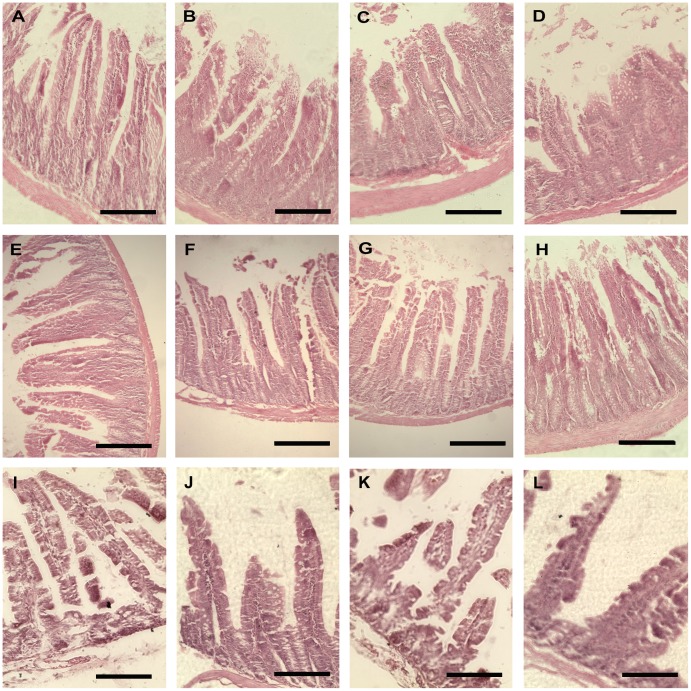
Effect of zearalenone (ZEN) on the jejunum histology in rats. Samples were collected from the pregnant dams (A–D) at gestation day 8, and weaned dams (E–H) and pups (I–L) at three weeks after born. The results were showed from left to right-ZEN0, ZEN50, ZEN100, ZEN150 in each period of the rats. (A)(E, F): jejunal histology demonstrated intact intestinal villi structure. (B) (G): mild local disruption of villus tips. (C) (H): denudated villi with partial digestion with moderate to massive epithelial lifting. (D): villi loss and disruption in integrity of villi. (A–H): HE, 200×; (I–L) HE, 400×. ZEN 0, ZEN 50, ZEN 100, and ZEN 150 means a daily feeding with ZEN at doses of 0.3, 48.5, 97.6, and 146.0 mg/kg respectively, at gestation days 1 to 7.

ZEN damaged the villous structure in a dose-dependent manner in the jejunum of pregnant dams. The ZEN0 group showed normal intestinal morphology. Mild local disruptions of villus tips were observed in the ZEN50 group. Most regions of the jejunum showed denudated villi with part digestion in the ZEN100 group. In the group of ZEN150, villi loss, disruption in integrity of villi were commonly observed. The intestinal mucosal structure could be partially recovered in the ZEN50, ZEN100 groups of the dams at weaning. However, in the ZEN150 group, jejunal sections still showed mild disruption of villi. Histological examination of the pups groups revealed normal mucosal structure and exhibited structural integrity (Figure 6).

By examining the microstructure of the intestinal mucosa, we found that ZEN affected villus and crypt structures. In the jejunum of pregnant rats, villus height was dose-dependently decreased in the ZEN-treated groups, and the ZEN150 group exhibited a significantly lower villus height compared with the ZEN0 group (P<0.05). The jejunal crypt depth was increased in the ZEN-treated groups, but the changes were only significant in the ZEN50 group (P<0.05). The villus height in the jejunum of weaned dams were significantly decreased in the ZEN150 group compared with other groups (P<0.05), whereas no differences were observed in the crypt depth. For the weaned pups, neither the villus height nor the crypt depth was affected by the treatment of female rats with ZEN (Table 3).

**Table 3:** Effect of zearalenone (ZEN) on villus hight and crypt depth of the jejunum in pregnant dams at gestation day 8, and weaned dams and pups at three weeks after born (µm).

	ZEN0	ZEN50	ZEN100	ZEN150
Pregnant dams				
Villus	245.80±9.82^a^	224.00±5.31^ab^	209.74±10.92^ab^	197.33±13.75^b^
Crypt	136.79±12.86^a^	167.01±4.72^b^	154.28±5.21^ab^	141.56±9.59^ab^
Weaned dams				
Villus	233.89±12.48^b^	233.16±6.17^b^	231.68±5.63^b^	218.19±7.86^a^
Crypt	131.816±11.48	138.08±4.71	131.21±7.83	139.42±6.76
Weaned pups				
Villus	97.24±4.01	96.548±2.04	97.79±3.97	97.37±4.05
Crypt	55.80±3.26	56.238±1.22	57.04±4.03	55.67±1.14

All of the values are expressed as the means ±SEM (n = 12).

ZEN 0, ZEN 50, ZEN 100, and ZEN 150 means a daily feeding with ZEN at doses of 0.3, 48.5, 97.6, and 146.0 mg/kg respectively, at gestation days 1 to 7.

a, b, c means within a row with no common superscripts differ significantly (P<0.05).

## Discussion

Early pregnancy is a sensitive period that can be influenced by ZEN exposure which affects early pregnancy events, including fertilization, embryo development, embryo transport, and embryo implantation [54]. Important developmental changes take place among early gestational days, when the primitive yolk sac placenta is replaced by the chorioallantoic placenta [55]. The chorioallantoic placenta is vascularized by allantoic vessels and becomes more permeable [56]. Time that ZEN exists in the body could be extended due to the extensive enterohepatic cycling [6], [57]. Thus, a high dose of the toxin could transfer to the embryos.

An important function of intestinal epithelia is to provide a barrier against the penetration of food contaminants and pathogens present in the intestinal lumen. The disruption of the intestinal barrier induced increased penetration of the normally excluded luminal substances that could promote intestinal disorders [41], [58]. Studies about the effects of ZEN on the intestine have focused on combinational action with other *Fusarium* mycotoxins, such as deoxynivalenol, T-2, or fumonisin [15], [59]–[61], while few studies have investigated the *in vivo* effects of ZEN on the intestine. The present investigation was conducted to study the toxic effects of maternal ZEN exposure on antioxidant status, immune response, expression of junction proteins, and villous structure in the intestinal samples of pregnant rats. These indexes were also examined in the jejunal samples of weaned offspring to investigated the effects of ZEN on intestinal development of the pups during the fetal period.

Following ingestion of food or feed, ZEN is absorbed from the upper part of intestinal tract. ZEN is extensively absorbed after its oral administration in rats, rabbits and humans [17]. We detected ZEN residues in all of the jejunal samples, and found no significant amounts of ZEN, indicating the rapid absorption of ZEN in jejunum.

Several studies both *in vitro* and *in vivo* reported that ZEN enhanced the formation of reactive oxygen species (ROS) and caused oxidative damage [22]–[25], [62]. Oxidative stress results in damage to cellular structures and has been linked to many diseases [63]. MDA is the end product of lipoperoxidation and is considered as an excellent index of lipid peroxidation [64], [65]. As showed by previous research, the concentrations of MDA were significantly increased in the liver, kidney, and testis of Balb/c mice treated with ZEN 40 mg/kg bw [25], [66]. Our results showed that the MDA concentrations were significantly increased in the ZEN-treated group of the pregnant dams and in the ZEN150 group of weaned dams, indicating the presence of oxidative stress in the jejunum. The variational trend of SOD activity was opposite to MDA with the increase of ZEN in the pregnant and weaned dams. The activity of SOD is known to serve protective function for the elimination of reactive free radicals and thus it represents an important antioxidant defense in nearly all cells exposed to oxygen [67], [68]. The finding in our result may be due to the increase of ROS in the jejunal tissue. CAT as an early marker of oxidative stress exhibited a decreasing tendency of activity in pregnant dams, which supports the hypothesis.

GPx can modify the poisonous peroxide to a non-toxic hydroxyl compound in order to protect the membrane structure and function. The increased GPx activity was observed in all of the ZEN-treated groups of pregnant dams, which may be due to the defending function of the organism itself against the ROS generation induced by ZEN. The mRNA expression of GPx2 was increased in the ZEN-treated groups of pregnant dams and the ZEN150 group of weaned dams. In addition, GPx2 mRNA was increased in the ZEN100 and ZEN150 groups of weaned pups. The up-regulated expression of GPx2 has been reported as an intestinal defending function against hydroperoxide absorption [69], which may explain the similar results of the MDA, SOD, GPx between the ZEN100 and ZEN150 groups of the pregnant dams. Results in weaned pups indicated the existence of oxidative stress in the jejunum. Other studies have demonstrated that ZEN induced increased activity of GPx in the duodenum mucosa of chicken [70] and increased mRNA expression of GPx in the liver of piglets [23] which was similar to our results. Antioxidant enzymes are considered to be the first line of cellular defense against oxidative damage. Based on the tendency of the antioxidant enzyme activity exhibited in the study, the oxidative stress damage caused by ZEN in the pregnant dams was further confirmed and in a dose-dependent manner. We have observed that the oxidative stress caused by ZEN during early pregnancy in the ZEN50 and ZEN100 groups of weaned dams could be recovered. However, the up-regulated MDA level and GPx activity and down-regulated SOD activity in ZEN150 group of weaned dams suggested the long-term oxidative stress caused by the high dose of ZEN, and the oxidative stress was unrecoverable even through the defending function of the intestine.

Oxidative stress and inflammation are tightly correlated. Lipid peroxidation may bring about protein damage either through direct attack by free radicals or through chemical modification by its end products, e.g. MDA [71]. Pathways that generate mediators of inflammation (e.g., adhesion molecules, and interleukins) are induced by oxidative stress [72], [73]. It is generally accepted that intestinal epithelium, as the interface between the highly antigenic luminal environment and the mucosal immune system, plays an active role in the immune responsiveness of intestinal mucosa. Immune responses in the intestinal mucosa are partly controlled by cytokines release in response to environmental stimuli. In the present study, we investigated the effects of different doses of ZEN on the production of pro-inflammatory cytokines (IL-1α, IL-1β, IL-6, IL-8 and TNF-α) in the jejunum, which are considered to play a key role in the regulation of the immune and inflammatory responses. ZEN has been described as either an inductor or a suppressor of pro-inflammatory cytokines [26], [74], [75]. In our result, the expression of IL-1β, IL-6, IL-8 and TNF-α in gene level exhibited a decreased tendency in pregnant dams. However, the expression of IL-1α mRNA was up-regulated in pregnant dams. The concentrations of cytokines were consistent with the changes in gene levels to a large extent, but not dose-related. Cytokine assays are not sufficiently sensitive to detect minute amounts of cytokines secreted in potential target tissues *in vivo*, and the adsorption and uptake of cytokines may impair the accurate quantitation of secreted cytokines [76], which may explain the different response of cytokines in protein and gene levels. The results of the inhibition of cytokine secretions were similar to those of Marin et al., who demonstrated that ZEN depressed the inflammatory cytokine secretions (TNF-a and IL-1β) both in the PBMCs at 5 and 10 µM of ZEN and in the liver of weanling piglets treated with 250ppb of ZEN [23], [75]. On the other hand, it has been shown that 40 µM ZEN could significantly increase the mRNA expression of IL-1α in porcine jejunal epithelial cell line [61].

As one of the pro-inflammatory cytokines produced in intestinal mucosal cells, IL-8 could enhance cell proliferation and control the repair processes during injury of the intestinal mucosa or cytotoxic stress [77], [78]. Because of its impact on the constitutive synthesis of IL-8, ZEN could perturb the maintenance of the steady homeostasis and the healing properties of the intestine. The weaned pups in the ZEN100 and ZEN150 groups exhibited a significant decrease in IL-8 expression, indicating the unrecoverable immune disorder caused by maternal ZEN. It has been established that the intestine has its own immune network, which causes localized induction of various cytokines and chemokines [79]. In our study, the divergent changes in inflammatory response indicate that ZEN induce immunotoxic effects in the jejunum, which impacted the capacity of the organism both to eradicate injurious stimuli and to initiate healing process.

Nrf2 is reported to be the key transcription factor that regulates cellular antioxidant response and counteracts inflammation [80]–[82]. The Nrf2-induced mechanism is activated by the stimulation of invading pathogens, and then Nrf2 was translocated from the cytoplasm to the nucleus, through its nuclear localization sequence. Up-regulated expression of the Nrf2 could activate the expression of downstream antioxidant genes (such as GPx2), reduce the inflammatory response and ameliorate the intestine damage [83]. In the present study, dose-related increase of Nrf2 expression was observed in pregnant dams. The results suggested that the expression of Nrf2 was activated with the ZEN ingestion in the jejunum. We also observed the increased expression of Nrf2 in the ZEN-treated groups of weaned dams. Considering the results of the normal level of cytokines and antioxidant capacity in the ZEN50 and ZEN100 groups of weaned dams, we believe that the increased Nrf2 is one of the ways to ameliorate the intestine damage through regulating the cellular antioxidant response and counteracting inflammation. However, in the ZEN150 group of weaned dams, the damage caused by ZEN was irreversible.

Intestinal barrier function is affected by the expression of the tight-junction and gap-junction proteins. In our study, the mRNA expression of tight junction proteins showed a strong correlation with the concentrations of ZEN in pregnant dams. To the best of our knowledge, this study provided the first demonstration that the ZEN-contaminated diet reduced mRNA expression of occludin and claudin-4. The claudin-4 and occludin are important for the formation of the actual tight junction seal in intestinal epithelium, which is involved in gut barrier function. The reduction of claudin-4 and occludin suggests defective adhesive properties in enterocytes that would correlate with an increased intestinal translocation of toxic luminal antigens, promoting intestinal inflammation [84]. Except the tight junctions, gap junctions exist between adjacent cells, allowing the transfer of small molecules (under 1000 daltons). The intestinal epithelial cells are physically and functionally interconnected via membrane channels that are composed of the gap junction Cx43 regulating their migration [85]. ZEN was found to inhibit gap junction intercellular communications [86]. As the results showed, the expression of Cx43 was decreased in a dose-dependent manner in pregnant dams. Changes were not recovered in the weaned dams of the ZEN150 group. Previous studies have shown that Cx43 plays an important role in innate immune control of commensal-mediated intestinal epithelial wound repair [87]. So the data are also confirmed by the results that ZEN induced oxidative stress and caused inflammation in the pregnant dams. Decreased levels of TNF-a and IL-1β observed in the jejunum could also contribute to tight-junction barrier defects [88], [89]. The down-regulated expression of junction proteins could increase intestinal barrier permeability, resulting in damage of the intestinal barrier function in rat.

The decreased expression of junction proteins induced by other toxins is always followed by the changes of intestinal villous structure [90], [91]. In addition, hypersensitivity to antigens in diet could induce morphological changes in the intestine [92]. However, there are few studies about the effects of ZEN on the intestinal morphology which is a commonly indicator of intestinal health in the studies focusing on the influence of food-derived antigens. The main histological changes observed in pregnant dams included the decreased villus height (ZEN150), the increased crypt depth (ZEN50). We also observed that pregnant rats exhibited mild to moderate intestinal lesions, including the detachment of the intestinal epithelial cells and the denudation of villi with part digestion. Damage was not recovered in the high-dose ZEN groups of weaned dams. Intestinal epithelial cells act as a barrier against the penetration of microbial pathogens, cytotoxic agents, and other intestinal contents [93]. Once the jejunum intestinal epithelial cells shed and microvillus height became shorter and sparser, the permeability increased, which then augmented inflammatory and amplified disturbances in gut motor [94]. The histopathological changes observed in the present study indicate that the intestine is inflamed or damaged as a result of ZEN. Possible explanation for these histological changes can be a direct irritant effect of ZEN or suppression of mitosis or protein synthesis [22], [95]. However, the normal mucosal appearance and junction proteins in the pups indicated the intestinal structure was not affected by maternal ZEN treatment.

As one of the natural estrogen-like molecules present in our daily environment, ZEN can represent an important exposure source at critical times, such as early gestation. The early events of pregnancy are associated with rapid changes in the expression of genes required for nutrient transport, cellular remodeling, angiogenesis, and relaxation of vascular tissues, as well as cell proliferation and migration [96]. Maternal effects can impact the morphology, physiology and behavior in future generations and even last a lifetime. Previous work have proposed that ZEN exposure during early gestation induced teratogenesis in the fetuses [10]. In the present study, we have observed the down-regulated expression of pro-inflammatory IL-8 and the up-regulated GPx2 mRNA in the jejunum of weaned pups. We considered that these deleterious effects in pups caused by maternal ZEN exposure, lasted from the stages of pregnancy to weaning.

## Conclusions

The toxic effects of the ZEN exposure on the intestinal function of pregnant dams are summarized in four aspects: causing oxidative stress, inducing inflammation, altering villous structure, and impairing intestinal barrier function. These changes are in a dose-dependent manner in pregnant dams and are unrecovered to some extent in the ZEN100 and ZEN150 groups of weaned dams. The expression of IL-8 and GPx2 in the pups were affected by the maternal exposure to ZEN100 and ZEN150, indicating the toxic effects of high-dose ZEN on the intestinal development during early pregnancy. We also found that the activation of Nrf2 was related to the jejunal antioxidant status and the inflammatory response, and the Nrf2-mediated signal pathway was one of mechanism that ZEN mediated toxicity in the jejunum.

## References

[1] Fink-Gremmels J (1999) Mycotoxins: their implications for human and animal health. Vet Quart 21: 115–120. 10.1080/01652176.1999.9695005 10568000

[2] Oswald IP, Marin DE, Bouhet S, Pinton P, Taranu I, Accensi F (2005) Immunotoxicological risk of mycotoxins for domestic animals. Food Addit Contam 22: 354–360. 16019805 10.1080/02652030500058320

[3] Richard JL (2007) Some major mycotoxins and their mycotoxicoses-an overview. Int J Food Microbiol 119: 3–10. 17719115 10.1016/j.ijfoodmicro.2007.07.019

[4] Tabuc C, Marin D, Guerre P, Sesan T, Bailly JD (2009) Molds and mycotoxin content of cereals in southeastern. Romania J Food Prot 72: 662–665. 19343960 10.4315/0362-028x-72.3.662

[5] Etienne M, Dourmad JY (1994) Effects of zearalenone or glucosinolates in the diet on reproduction in sows: a review. Livest Prod Sci: 99–113.

[6] Appelgren LE, Arora RG, Larsson P (1982) Autoradiographic studies of ^3^H zearalenone in mice. Toxicology 25: 243–253. 6218655 10.1016/0300-483x(82)90033-6

[7] Etienne M, Jemmali M (1982) Effects of zearalenone (F2) on estrous activity and reproduction in gilts. J Anim Sci 55: 1–10. 10.2527/jas1982.5511 6214538

[8] Diekman MA, Long GG (1989) Blastocyst development on days 10 or 14 after consumption of zearalenone by sows on days 7 to 10 after breeding. Am J Vet Res 50: 1224–1227. 2528931

[9] Young LG, Ping H, King GJ (1990) Effects of feeding zearalenone to sows on rebreeding and pregnancy. J Anim Sci 68: 15–20. 2137439 10.2527/1990.68115x

[10] Zhang Y, Jia Z, Yin S, Shan A, Gao R, et al. (2013) Toxic effects of maternal zearalenone exposure on uterine capacity and fetal development in gestation rats. Reprod Sci 1933719113512533. 10.1177/1933719113512533 PMC4016724 24357638

[11] Dänicke S, Brüssow KP, Goyarts T, Valenta H, Ueberschär KH, et al (2007) On the transfer of the Fusarium toxins deoxynivalenol (DON) and zearalenone (ZON) from the sow to the full-term piglet during the last third of gestation. Food Chem Toxicol 45: 1565–1574. 17399880 10.1016/j.fct.2007.02.016

[12] Schnurrbusch U, Heinze A (2002) Achtung Mykotoxine. Tierhaltung 10: 112–117.

[13] Tiemann U, Dänicke S (2006) In vivo and in vitro effects of the mycotoxins zearalenone and deoxynivalenol on different non-reproductive and reproductive organs in female piglets. Food Addit Contam 24: 306–314. 10.1080/02652030601053626 17364934

[14] Soderholm JD, Perdue MH (2001) Stress and gastrointestinal tract. II. Stress and intestinal barrier function. Am J Physiol Gastrointest Liver Physiol 280: G7–13. 11123192 10.1152/ajpgi.2001.280.1.G7

[15] Bouhet S, Oswald IP (2005) The effects of mycotoxins fungal food contaminants on the intestinal epithelial cell-derived innate immune response. Vet Immunol Immunop 108: 199–209. 10.1016/j.vetimm.2005.08.010 16144716

[16] Videmann B, Mazallon M, Tep J, Lecoeur S (2008) Metabolism and transfer of the mycotoxin zearalenone in human intestinal Caco-2 cells. Food Chem Toxicol 46: 3279–3286. 18692541 10.1016/j.fct.2008.07.011

[17] Kuiper-Goodman T, Scott PM, Watanabe H (1987) Risk assessment of the mycotoxin zearalenone. Regul Toxicol Pharmacol 7: 253–306. 2961013 10.1016/0273-2300(87)90037-7

[18] Maaroufi K, Chekir L, Creppy EE, Ellouz F, Bacha H (1996) Zearalenone induces modifications of haematological and biochemical parameters in rats. Toxicon 34: 535–540. 8783448 10.1016/0041-0101(96)00008-6

[19] Zinedine A, Soriano JM, Molto JC, Manes J (2007) Review on the toxicity occurrence metabolism detoxification regulations and intake of zearalenone: an oestrogenic mycotoxin. Food Chem Toxicol 45: 1–18. 17045381 10.1016/j.fct.2006.07.030

[20] Kim IH, Son HY, Cho SW, Ha CS, Kang BH (2003) Zearalenone induces male germ cell apoptosis in rats. Toxicology Letters 138: 185–192. 12565195 10.1016/s0378-4274(02)00405-8

[21] Pfohl-Leszkowicz A, Chekir-Ghedira L, Bacha H (1995) Genotoxicity of zearalenone an oestrogenic mycotoxin: DNA adductsformation in female mouse tissues. Carcinogenesis 16: 2315–2320. 7586128 10.1093/carcin/16.10.2315

[22] Abid-Essefi S, Ouanes Z, Hassen W, Baudrimont I, Creppy EE, et al (2004) Cytotoxicity inhibition of DNA and protein syntheses and oxidative damage in cultured cells exposed to zearalenone. Toxicol In Vitro 18: 467–474. 15130604 10.1016/j.tiv.2003.12.011

[23] Marin DE, Pistol GC, Neagoe IV, Calin L, Taranu I (2013) Effects of zearalenone on oxidative stress and inflammation in weanling piglets. Food Chem Toxicol 58: 408–415. 23727178 10.1016/j.fct.2013.05.033

[24] Jia Z, Liu M, Qu Z, Zhang Y, Yin S, Shan A (2014) Toxic Effects of zearalenone on oxidative stress, inflammatory cytokines, biochemical and pathological changes induced by this toxin in the kidney of pregnant rats. Environ Toxicol Phar 37: 580–591. 10.1016/j.etap.2014.01.010 24562056

[25] Salah-Abbès JB, Abbès S, Abdel-Wahhab MA, Oueslati R (2009) Raphanus sativus extract protects against zearalenone induced reproductive toxicity oxidative stress and mutagenic alterations in male Balb/c mice. Toxicon 53: 525–533. 19673099 10.1016/j.toxicon.2009.01.013

[26] Salah-Abbès JB, Abbès S, Houas Z, Abdel-Wahhab MA, Oueslati R (2008) Zearalenone induces immunotoxicity in mice: Possible protective effects of radish extract (Raphanus sativus). J Pharm Pharmacol 60: 761–770. 18498713 10.1211/jpp.60.6.0012

[27] Pestka JJ, Tai JH, Witt MF, Dixon DE, Forsell JH (1987) Suppression of immune response in the B6C3F1 mouse after dietary exposure to the Fusarium mycotoxins deoxynivalenol (vomitoxin) and zearalenone. Food Chem Toxicol 25: 297–304. 2953660 10.1016/0278-6915(87)90126-8

[28] Luongo D, De Luna R, Russo R, Severino L (2008) Effects of four Fusarium toxins (fumonisin B(1) alpha-zearalenol nivalenol and deoxynivalenol) on porcine whole-blood cellular proliferation. Toxicon 52: 156–162. 18620720 10.1016/j.toxicon.2008.04.162

[29] Abid-Essefi S, Baudrimont I, Hassen W, Ouanes Z, Mobio TA, et al (2003) DNA fragmentation apoptosis and cell cycle arrest induced by zearalenone in cultured DOK Vero and Caco-2 cells: prevention by Vitamin E. Toxicology 192: 237–248. 14580790 10.1016/s0300-483x(03)00329-9

[30] Kouadio JH, Mobio TA, Baudrimont I, Moukha S, Dano SD, et al (2005) Comparative study of cytotoxicity and oxidative stress induced by deoxynivalenol zearalenone or fumonisin B1 in human intestinal cell line Caco-2. Toxicology 213: 56–65. 16019124 10.1016/j.tox.2005.05.010

[31] Marin DE, Taranu I, Pistol G, Stancu M (2013) Effects of zearalenone and its metabolites on the swine epithelial intestinal cell line: IPEC 1. P Nutr Soc 72: E40.

[32] Wan LYM, Turner PC, El-Nezami H (2013) Individual and combined cytotoxic effects of Fusarium toxins (deoxynivalenol, nivalenol, zearalenone and fumonisins B1) on swine jejunal epithelial cells. Food Chem Toxicol 57: 276–283. 23562706 10.1016/j.fct.2013.03.034

[33] DeSesso JM, Jacobson CF (2001) Anatomical and physiological parameters affecting gastrointestinal absorption in humans and rats. Food Chem Toxicol 39: 209–228. 11278053 10.1016/s0278-6915(00)00136-8

[34] Ma Y, Semba S, Khan RI, Bochimoto H, Watanabe T, et al (2013) Focal adhesion kinase regulates intestinal epithelial barrier function via redistribution of tight junction. Biochim Biophys Acta 1832: 151–159. 23064287 10.1016/j.bbadis.2012.10.006

[35] Harhaj NS, Antonetti DA (2004) Regulation of tight junctions and loss of barrier function in pathophysiology. Int J Biochem Cell Biol 36: 1206–1237. 15109567 10.1016/j.biocel.2003.08.007

[36] Bruzzone R, White TW, Paul DL (1996) Connections with connexins: the molecular basis of direct intercellular signalling. Eur J Biochem 238: 1–27. 8665925 10.1111/j.1432-1033.1996.0001q.x

[37] Kumar NM, Gilula NB (1996) The gap junction communication channel. Cell 84: 381–388. 8608591 10.1016/s0092-8674(00)81282-9

[38] Goodenough DA (1975) The structure of cell membranes involved in intercellular communication. Am J Clin Pathol 63, 636–645. 10.1093/ajcp/63.5.636 1130322

[39] Li Z, Zhou Z, Daniel EE (1993) Expression of gap junction connexin 43 and connexin 43 mRNA in different regional tissues of intestine in dog. Am J Physiol 265: G911–G916. 8238521 10.1152/ajpgi.1993.265.5.G911

[40] Brandtzaeg P (1996) History of oral tolerance and mucosal immunitya. Ann NY Acad Sci 778: 1–27. 8610963 10.1111/j.1749-6632.1996.tb21110.x

[41] Oswald IP (2006) Role of intestinal epithelial cells in the innate immune defence of the pig intestine. Vet Res 37: 359–368. 16611553 10.1051/vetres:2006006

[42] Osselaere A, Santos R, Hautekiet V, De Backer P, Chiers K, et al (2013) Deoxynivalenol impairs hepatic and intestinal gene expression of selected oxidative Stress, tight junction and inflammation proteins in broiler chickens, but addition of an adsorbing agent shifts the effects to the distal parts of the small intestine. PloS one 8: e69014. 23922676 10.1371/journal.pone.0069014 PMC3724867

[43] Ruddick JA, Scott PM, Harwig J (1976) Teratological evaluation of zearalenone administered orally to the rat. B Environ Contam Tox 15: 678–681. 10.1007/BF01685617 938760

[44] Collins TF, Sprando RL, Black TN, Olejnik N, Eppley RM, et al (2006) Effects of zearalenone on in utero development in rats. Food Chem Toxicol 44: 1455–1465. 16797818 10.1016/j.fct.2006.04.015

[45] Arora RG, Frolen H, Nilsson A (1981) Interference of mycotoxins with prenatal development of the mouse I Influence of aflatoxin B1 ochratoxin A and zearalenone. Acta Vet Scand 22: 524–534. 6211964 10.1186/BF03548677 PMC8300444

[46] Bennett GA, Nelsen TC, Miller BM (1994) Enzyme-linked immunosorbent assay for detection of zearalenone in maize wheat and pig feed: Collaborative study. J AOAC Int 77: 1500–1509. 7819759

[47] Nuryono N, Noviandi CT, Böhm J, Razzazi-Fazeli E (2005) A limited survey of zearalenone in Indonesian maize-based food and feed by ELISA and high performance liquid chromatography. Food control 16: 65–71.

[48] Bloom RJ, Westerfe WW (1971) The thiobarbituric acid reaction in relation to fatty livers. Arch Biochem Biophys 145: 669–675. 5125212 10.1016/s0003-9861(71)80027-9

[49] Rongzhu L, Suhua W, Guangwei X, Chunlan R, Fangan H, et al (2009) Effects of acrylonitrile on antioxidant status of different brain regions in rats. Neurochem Int 55: 52–557. 19463879 10.1016/j.neuint.2009.05.009

[50] Elstner EF, Heupel A (1976) Inhibition of nitrite formation from hydroxylammonium-chloride: a simple assay for superoxide dismutase. Anal Biochem 70: 616–620. 817618 10.1016/0003-2697(76)90488-7

[51] Sedlak J, Lindsay RH (1968) Estimation of total protein-bound and nonprotein sulfhydryl groups in tissue with Ellman's reagent. Anal Biochem 25: 192–205. 4973948 10.1016/0003-2697(68)90092-4

[52] Aebi H (1984) Catalase in vitro. Meth Enzymol 105: 121–126. 6727660 10.1016/s0076-6879(84)05016-3

[53] Livak KJ, Schmittgen TD (2001) Analysis of relative gene expression data using real-time quantitative PCR and the 2^−ΔΔCt^ method. Methods 25: 402–408. 11846609 10.1006/meth.2001.1262

[54] Zhao F, Li R, Xiao S, Diao H, Viveiros MM, et al (2013) Postweaning exposure to dietary zearalenone a mycotoxin promotes premature onset of puberty and disrupts early pregnancy events in female mice. Toxicol Sci 132: 431–442. 23291560 10.1093/toxsci/kfs343 PMC3595522

[55] Beaudoin AR (1980) Embryology and teratology In: Baker HJ Lindsey JR Weisbroth SH editors. Academic Press 75–101.

[56] DeSesso JM (1997) Comparative Embryology In: Hood RD editors Handbook of Developmental Toxicology London: CRC Press 111–174.

[57] Biehl ML, Prelusky DB, Koritz GD, Hartin KE, Buck WB, et al (1993) Biliary excretion and enterohepatic cycling of zearalenone in immature pigs. Toxicol Appl Pharm 121: 152–159. 10.1006/taap.1993.1140 8337696

[58] Arrieta MC, Bistritz L, Meddings JB (2006) Alterations in intestinal permeability. Gut 55: 1512–1520. 16966705 10.1136/gut.2005.085373 PMC1856434

[59] Obremski K, Zielonka L, Gajecka M, Jakimiuk E, Bakuła T, et al (2007) Histological estimation of the small intestine wall after administration of feed containing deoxynivalenol, T-2 toxin and zearalenone in the pig. Pol J Vet Sci 11: 339–345. 19227132

[60] Kouadio JH, Dano SD, Moukha S, Mobio TA, Creppy EE (2007) Effects of combinations of Fusarium mycotoxins on the inhibition of macromolecular synthesis, malondialdehyde levels, DNA methylation and fragmentation, and viability in Caco-2 cells. Toxicon 49: 306–317. 17109910 10.1016/j.toxicon.2006.09.029

[61] Wan MLY, Woo CSJ, Turner PC, Wan JMF, Hani EN (2013) Individual and combined effects of Fusarium toxins on the mRNA expression of pro-inflammatory cytokines in swine jejunal epithelial cells. Toxicology letters, 200, 238–246. 10.1016/j.toxlet.2013.05.003 23688591

[62] Yu JY, Zheng ZH, Son YO, Shi X, Jang YO, et al (2011) Mycotoxin zearalenone induces AIF- and ROS-mediated cell death through p53- and MAPK-dependent signaling pathways in RAW2647 macrophages. Toxicol In Vitro 25: 1654–1663. 21767629 10.1016/j.tiv.2011.07.002

[63] Marin DE, Taranu I (2012) Overview on aflatoxins and oxidative stress. Toxin Rev 31: 32–43.

[64] Bird RP, Draper HH (1984) Comparative studies on different methods of malonaldehyde determination. Method Enzymol 105: 299. 10.1016/s0076-6879(84)05038-2 6727668

[65] Tomita M, Okuyama T, Kawai S (1990) Determination of malonaldehyde in oxidized biological materials by high-performance liquid chromatography. J Chromatogr A 515: 391–397. 10.1016/s0021-9673(01)89334-1 2283368

[66] Zourgui L, Golli EE, Bouaziz C, Bacha H, Hassen W (2008) Cactus (Opuntia ficus-indica) cladodes prevent oxidative damage induced by the mycotoxin zearalenone in Balb/C mice. J Food Chem Toxicol 46: 1817–1824. 10.1016/j.fct.2008.01.023 18313193

[67] Liska DJ (1998) The detoxification enzyme systems. Altern Med Rev 3: 187–198. 9630736

[68] Cheung CC, Zheng GJ, Richardson BJ, Lam PKS (2001) Relationship between tissue concentrations of polycyclic aromatic hydrocarbons and antioxidative responses of marine mussels. Aquat Toxicol 52: 189–203. 11239681 10.1016/s0166-445x(00)00145-4

[69] Brigelius-Flohé R (1999) Tissue-specific functions of individual glutathione peroxidases. Free Radic. Biol Med 27: 951–965. 10.1016/s0891-5849(99)00173-2 10569628

[70] Grešáková L,, Bořutová R, Faix Š, Plachá I, Čobanová K, et al (2012) Effect of lignin on oxidative stress in chickens fed a diet contaminated with zearalenone. Acta Vet Hung 60: 103–114. 22366136 10.1556/AVet.2012.009

[71] Halliwell B, Gutteridge JMC (1999) Free radicals in biology and medicine (3rd ed). Oxford: Clarendon Press.

[72] Jenny NS (2012) Inflammation in aging: cause effect or both? Discov Med 13: 451–460. 22742651

[73] Kim YJ, Kim EH, Hahm KB (2012) Oxidative stress in inflammation-based gastrointestinal tract diseases: challenges and opportunities. J Gastroenterol Hepatol 27: 1004–1010. 22413852 10.1111/j.1440-1746.2012.07108.x

[74] Ruh MF, Bi Y, Cox L, Berk D, Howlett AC, et al (1998) Effect of environmental estrogens on IL-1b promoter activity in a macrophage cell line. Endocrine 9: 207–211. 9867255 10.1385/ENDO:9:2:207

[75] Marin DE, Taranu I, Burlacu R, Manda G, Motiu M, et al (2011) Effects of zearalenone and its derivatives on porcine immune response. Toxicol In Vitro 25: 1981–1988. 21763767 10.1016/j.tiv.2011.06.022

[76] Azconaolivera JI, Ouyang Y, Murtha J, Chu FS, Pestka JJ (1995) Induction of cytokine mRNAs in mice after oral exposure to the trichothecene vomitoxin (deoxynivalenol): relationship to toxin distribution and protein synthesis inhibition. Toxicol Appl Pharm 133: 109–120. 10.1006/taap.1995.1132 7597700

[77] Maheshwari A, Lacson A, Lu W, Fox SE, Barleycorn AA, et al (2004) Interleukin-8/CXCL8 forms an autocrine loop in fetal intestinal mucosa. Pediatr Res 56: 240–249. 15181190 10.1203/01.PDR.0000133196.25949.98

[78] Zachrisson K, Neopikhanov V, Wretlind B, Uribe A (2001) Mitogenic action of tumour necrosis factor-alpha and interleukin-8 on explants of human duodenal mucosa. Cytokine 15: 148–155. 11554784 10.1006/cyto.2001.0917

[79] Stadnyk AW (2002) Intestinal epithelial cells as a source of inflammatory cytokines and chemokines. Can J Gastroenterol 16: 241–246. 11981577 10.1155/2002/941087

[80] Rangasamy T, Guo J, Mitzner WA, Roman J, Singh A, et al (2005) Disruption of Nrf2 enhances susceptibility to severe airway inflammation and asthma in mice. J Exp Med 202: 47–59. 15998787 10.1084/jem.20050538 PMC2212893

[81] Pan H, Wang H, Zhu L, Mao L, Qiao L, et al (2011) Depletion of Nrf2 enhances inflammation induced by oxyhemoglobin in cultured mice astrocytes. Neurochem Res 36: 2434–2441. 21833844 10.1007/s11064-011-0571-6

[82] Thimmulappa RK, Lee H, Rangasamy T, Reddy SP, Yamamoto M, Kensler TW, et al (2006) Nrf2 is a critical regulator of the innate immune response and survival during experimental sepsis. J Clin Invest 116: 984–995. 16585964 10.1172/JCI25790 PMC1421348

[83] Banning A, Deubel S, Kluth D, Zhou Z, Brigelius-Flohé R (2005) The GI-GPx gene is a target for Nrf2. Mol Cell Biol 25: 4914–4923. 15923610 10.1128/MCB.25.12.4914-4923.2005 PMC1140597

[84] Maresca M, Fantini J (2010) Some food-associated mycotoxins as potential risk factors in humans predisposed to chronic intestinal inflammatory diseases. Toxicon 56: 282–294. 20466014 10.1016/j.toxicon.2010.04.016

[85] Leaphart CL, Qureshi F, Cetin S, Li J, Dubowski T, et al (2007) Interferon-gamma inhibits intestinal restitution by preventing gap junction communication between enterocytes. Gastroenterology 132: 2395–2411. 17570214 10.1053/j.gastro.2007.03.029

[86] Ouanes-Ben Othmen Z, Essefi SA, Bacha H (2008) Mutagenic and epigenetic mechanisms of zearalenone: prevention by Vitamin E. World Mycotoxin J 1: 369–374.

[87] Ey B, Eyking A, Gerken G, Podolsky DK, Cario E (2009) TLR2 mediates gap junctional intercellular communication through connexin-43 in intestinal epithelial barrier injury. J Biol Chem 284: 22332–22343. 19528242 10.1074/jbc.M901619200 PMC2755956

[88] Ye D, Ma I, Ma TY (2006) Molecular mechanism of tumor necrosis factor-a modulation of intestinal epithelial tight junction barrier. Am J Physiol Gastrointest Liver Physiol 290: 496–504. 10.1152/ajpgi.00318.2005 16474009

[89] Al-Sadi R, Ye D, Dokladny K, Ma TY (2008) Mechanism of IL-1beta-induced increase in intestinal epithelial tight junction permeability. J Immunol 180: 5653–5661. 18390750 10.4049/jimmunol.180.8.5653 PMC3035485

[90] McLaughlin J, Padfield PJ, Burt JP, O'Neill CA (2004) Ochratoxin A increases permeability through tight junctions by removal of specific claudin isoforms. Am J Physiol-Cell Ph 287: C1412–1417. 10.1152/ajpcell.00007.2004 15229101

[91] Pinton P, Nougayrède JP, Del Rio JC, Moreno C, Marin DE, et al (2009) The food contaminant deoxynivalenol decreases intestinal barrier permeability and reduces claudin expression. Toxicol Appl Pharm 237: 41–48. 10.1016/j.taap.2009.03.003 19289138

[92] Li DF, Nelssen JL, Reddy PG, Blecha F, Hancock JD, et al (1990) Transient hypersensitivity to soybean meal in the early-weaned pig. J Anim Sci 68: 1790–1799. 2384373 10.2527/1990.6861790x

[93] Yu LCH (2005) SGLT-1-mediated glucose uptake protects intestinal epithelial cells against LPS-induced apoptosis and barrier defects: a novel cellular rescue mechanism? FASEB J 19: 1822–1835. 16260652 10.1096/fj.05-4226com

[94] Collins SM (2001) Stress and the Gastrointestinal Tract IV. Modulation of intestinal inflammation by stress: basic mechanisms and clinical relevance. American Journal of Physiology. Gastrointest Liver Physiol 280: G315–G318. 10.1152/ajpgi.2001.280.3.G315 11171612

[95] Aida EM, Hassanane MS, Alla ESAA (2001) Genotoxic evaluation for the estrogenic mycotoxin zearalenone. Reprod Nutr Dev 41: 79–89. 11368247 10.1051/rnd:2001114

[96] Bazer FW, Spencer TE, Johnson GA, Burghardt RC, Wu G (2009) Comparative aspects of implantation. Reproduction 138: 195–209. 19502456 10.1530/REP-09-0158

